# Efficacy and Safety of Stereotactic Radiosurgery in Patients With Large-Volume Meningiomas ≥10 cm³: A Systematic Review and Single-Arm Meta-Analysis

**DOI:** 10.7759/cureus.99713

**Published:** 2025-12-20

**Authors:** José Eduardo Trejo Elías, André Méndez Bolaños Cacho, Andrés Márquez Luján, José Trejo López

**Affiliations:** 1 Department of Medicine, Universidad Panamericana, Mexico City, MEX; 2 Neurological Surgery, Hospital Médica Avanzada Contigo (MAC), Celaya, MEX

**Keywords:** adverse radiation effects, large-volume meningiomas, meta-analysis, stereotactic radiosurgery, treatment safety, tumor control

## Abstract

Large-volume intracranial meningiomas (LVMs) pose significant surgical challenges and are associated with increased morbidity despite advances in neurosurgical techniques. While stereotactic radiosurgery (SRS) is an established treatment for small- to medium-sized meningiomas, its efficacy and safety profile in LVMs (≥10 cm³) remain uncertain. We performed a systematic review and meta-analysis evaluating the efficacy and safety of SRS in patients with LVMs. PubMed, Embase, Web of Science, and Cochrane databases were searched for studies reporting outcomes of SRS in LVMs. Heterogeneity was assessed using the I² statistic, and pooled estimates were calculated using a random-effects model. Fifteen studies comprising 1,093 patients were included, of whom 955 (87%) presented with meningiomas measuring ≥10 cm³. The median follow-up duration across studies was 55 months (range of medians: 22-106 months). The overall tumor control rate was 91.2% (95% CI: 87.7-93.8%). Subgroup analyses demonstrated higher tumor control rates with hypofractionated (94.9%; 95% CI: 90.8-97.2%) compared to single-session (89.0%; 95% CI: 83.6-92.7%) and staged-SRS (82.6%; 95% CI: 68.9-91.1%). Progression-free survival at three and five years was 91.3% (95% CI: 84.6-95.2%) and 90.6% (95% CI: 82.2-95.3%), respectively. Adverse radiation effects occurred in 15.9% (95% CI: 7.8-29.9%), and symptomatic peritumoral edema in 4.7% (95% CI: 1.6-13.1%). In patients with LVMs, SRS is associated with high tumor control rates and an acceptable safety profile. Hypofractionated SRS appears to offer higher tumor control compared to single-session and staged approaches. Further prospective randomized trials are warranted to validate these findings and optimize treatment protocols.

## Introduction and background

Intracranial meningiomas are typically slow-growing, extra-axial tumors arising from the meninges and represent the most common primary intracranial neoplasm in adults. While many meningiomas can be managed surgically, larger lesions pose particular challenges due to mass effect, proximity to critical neurovascular structures, and increased operative morbidity. Although most meningiomas are histologically benign, clinical behavior and treatment-related risk are strongly influenced by tumor size and anatomic location, particularly when lesions abut or encase critical neurovascular structures. Stereotactic radiosurgery (SRS), which delivers highly conformal, high-dose radiation in one or a limited number of sessions, differs fundamentally from conventional fractionated radiotherapy by prioritizing spatial precision over dose fractionation. These characteristics make SRS well-suited for selected lesions but also raise important safety considerations as tumor volume increases.

In this context, SRS provides a minimally invasive treatment option for patients with intracranial meningiomas who are poor surgical candidates or have residual or recurrent disease following resection. While its efficacy and safety are well established for small- to medium-sized tumors, its use in large-volume meningiomas remains limited due to an increased risk of radiation-induced toxicity, including peritumoral edema and cranial neuropathies [1-3]. To mitigate these complications, alternative approaches, such as hypofractionated and staged-SRS, have been introduced [4].

A recent systematic review and meta-analysis [5] evaluated SRS for large intracranial meningiomas, defined broadly as tumors exceeding 8 cm³ or 2.5 cm in maximum diameter. Although this study reported encouraging local control rates and manageable complication profiles, its findings may not fully capture the risk profile of patients harboring significantly larger tumors. In contrast, our analysis specifically targets a higher-risk patient population by employing stricter inclusion criteria, focusing exclusively on tumors ≥10 cm³ and encompassing a broader patient population.

In response to this critical research gap, and in complement to the insights provided by prior studies [5,6], the present systematic review and meta-analysis rigorously assesses existing evidence on the efficacy and safety of SRS--administered either as primary or adjuvant therapy--in the management of large-volume intracranial meningiomas (≥10 cm³). This analysis emphasizes key clinical endpoints, including tumor control, the need for retreatment or surgical salvage, and the incidence of adverse radiation effects, with the aim of complementing and refining the existing evidence to better inform management strategies in this high-risk patient population.

## Review

Methods

This systematic review and meta-analysis follows the Cochrane Collaboration and the Preferred Reporting Items for the Systematic Reviews and Meta-analysis (PRISMA) statement guidelines to ensure comprehensive and transparent reporting [7,8]. The protocol was prospectively registered on PROSPERO under protocol CRD420251113947. 

Search strategy and data extraction 

We systematically searched PubMed, Web of Science, Embase, and the Cochrane Library (CENTRAL) from inception to July 29, 2025, using the following search strategy: (meningioma) AND (LVMs OR large) AND (SRS OR “stereotactic radiosurgery” OR “Gamma Knife” OR “linear accelerator-based radiosurgery” OR LINAC OR “CyberKnife” OR “staged radiosurgery” OR “volume staging” OR “dose-staging”). Two authors (J.T. and A.M.L.) independently conducted the literature search according to predefined eligibility criteria. Disagreements were resolved by consensus or adjudication by a third author (A.M.B.).

Data extraction was independently performed by two authors (J.T. and A.M.B.) using a standardized data collection form. Extracted variables included study characteristics (first author, year of publication, study design, and median follow-up), patient demographics and tumor features (sex, age, tumor volume, location, WHO grade, and prior surgical intervention), treatment parameters (SRS modality, number of fractions, and prescribed dose), and the number of events for each outcome of interest. Any discrepancies were resolved through discussion or, when necessary, by a third reviewer (A.M.L.). No imputation was performed for missing data.

Eligibility criteria

Inclusion in this meta-analysis was restricted to studies that met all the following eligibility criteria: randomized controlled trials or observational studies; (2) including patients with large-volume intracranial meningiomas (≥10 cm^3^); (3) treated with single-session SRS, hypofractionated SRS (delivered in two to five fractions with ≥5 Gy per fraction) or staged-SRS; (4) reporting the outcomes of interest. Exclusion criteria included: (1) studies with missing data on interventional therapy used; (2) studies that did not report outcomes separately for patients with meningioma stratified by tumor volume (≥10 cm³); (3) studies with overlapping populations; (4) conference abstracts or any reports not published in full. 

Outcomes of interest/endpoints 

The primary efficacy outcome was overall tumor control, defined as evidence of either stable disease or tumor regression on follow-up imaging. Secondary efficacy outcomes included progression-free survival (PFS) at three and five years, the need for repeat radiation therapy, and the need for surgical intervention, defined as salvage craniotomy with tumor resection due to either progressive tumor growth or peritumoral edema unresponsive to medical management.

Safety outcomes included the incidence of adverse radiation effects (AREs), symptomatic peritumoral edema, all-cause mortality, and tumor-related mortality.

Quality assessment and risk of publication bias assessment

For each included study, we assessed the risk of bias using the Risk of Bias in Non-randomized Studies of Interventions (ROBINS-I) tool [9]. Two authors (J.T. and A.M.B.) independently performed the assessment, and any disagreements were resolved by consensus following discussion of the underlying discrepancies. Publication bias was evaluated both visually by funnel plot inspection and statistically using Egger’s test. 

Statistical analysis 

Statistical analyses were performed using the meta package in R (version 4.5.0) (R Foundation for Statistical Computing, Vienna, Austria) [10] and RStudio (version 2024.12.1) (RStudio, PBC, Boston, MA) [11]. Proportions were pooled using metaprop() with a binomial-normal generalized linear mixed model (GLMM) (logit link; random intercept for study) under random effects; zero-event studies were retained without continuity correction. Heterogeneity was assessed using Cochran's Q test and the I² statistic. According to the Cochrane Handbook [7], I² values below 40% were considered potentially unimportant, whereas higher values indicated increasing heterogeneity. 

Subgroup analyses by stereotactic radiosurgery (SRS) modality-single-session, hypofractionated, and staged-were restricted to studies reporting outcomes from homogeneous treatment cohorts. Studies with mixed-modality populations or unstratified data were excluded to minimize confounding and maintain internal validity. Leave-one-out sensitivity analyses were performed for primary outcomes and those with high heterogeneity to assess the influence of individual studies on the pooled estimates. To examine whether anatomic location influenced radiosurgical toxicity, we conducted a univariable random-effects meta-regression modeling the association between the study-level proportion of supratentorial meningiomas and the rate of adverse radiation effects (AREs). Only studies explicitly reporting tumor location distribution were eligible. A p-value < 0.05 was considered statistically significant for all analyses.

Results

Study Selection 

**Figure 1:**
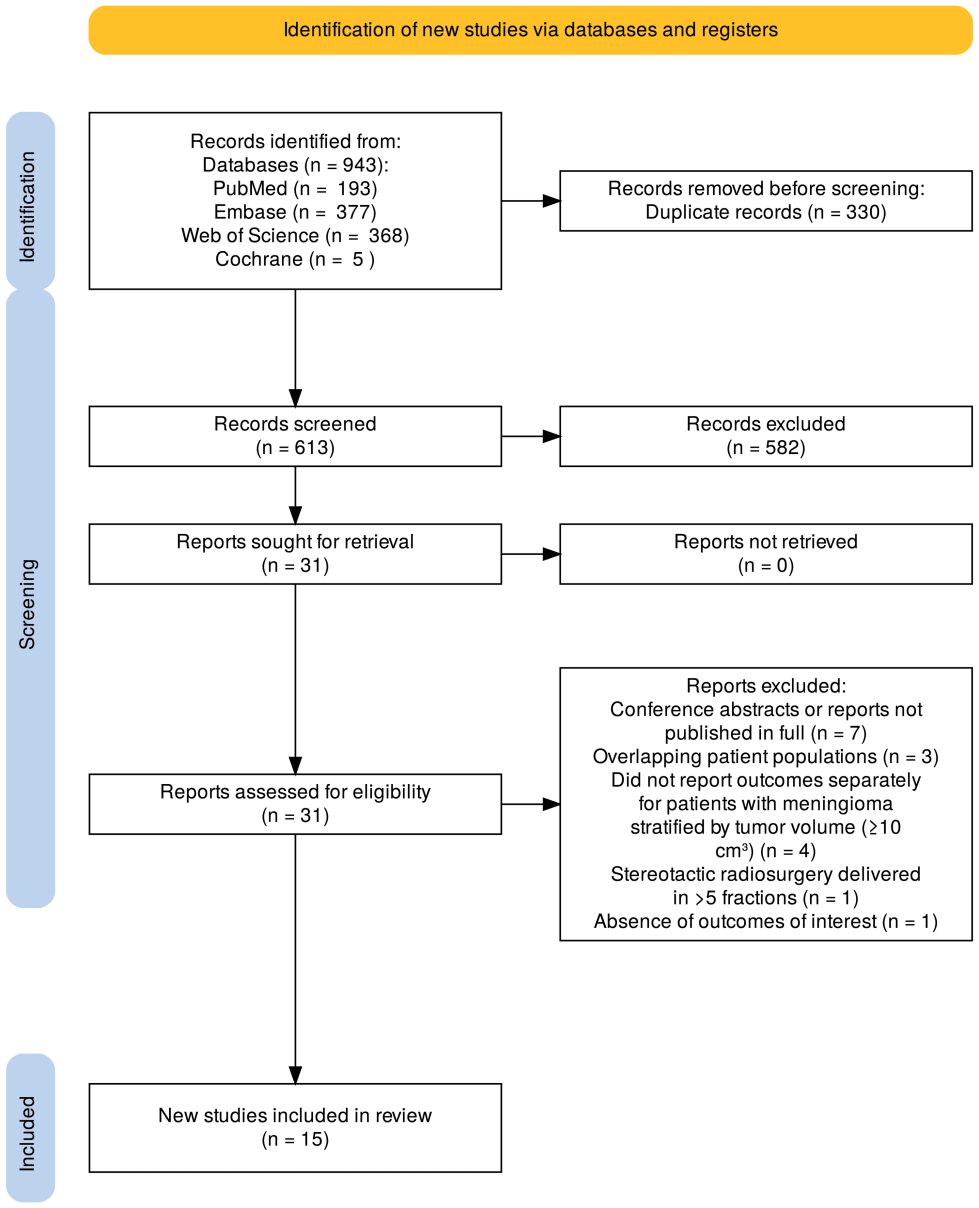
Preferred Reporting Items for Systematic Reviews and Meta-Analyses (PRISMA) flow diagram.

We identified 943 results in the initial database search (Figure 1). After removing duplicate records and screening by titles and abstracts, 31 studies were fully reviewed. A total of 15 studies were included in the final analysis, comprising 1,093 patients, of whom 955 (87%) presented with meningiomas measuring ≥10 cm³ treated with stereotactic radiosurgery. The median follow-up duration across studies was 55 months (range of medians: 22-106 months), and the number of patients with at least one previous surgery was 429 (39%). Other characteristics of the included studies are shown in Table 1. 

**Table 1:** Baseline characteristics of included studies Values are presented as n (%) or median (range), unless otherwise noted. *Certain values correspond to the entire study population and are not restricted to patients with large-volume meningiomas (>10 cm³). †Mean (SD). ‡Mean (range). Han's 2017 study appears twice because the authors reported baseline characteristics and outcomes separately for SS and HSRS cohorts. FX: fractions; HSRS: hypofractionated stereotactic radiosurgery; LVMs: large-volume meningiomas; SI: stage I; SII: stage II; SRS: stereotactic radiosurgery; SS: single-session; WHO 1: World Health Organization grade 1.

Study	Total sample size, n	LVMs, n (%)	Study design	Follow-up, months	SRS type	Dose, Gy	Fractions, n	Female, n (%)	Age, years	Volume, cm^3^	WHO 1, n (%)	Prior surgery, n (%)	Basal, n (%)
Bledsoe et al. 2010 [12]	116	116 (100%)	Retrospective cohort	70.1	SS-SRS	15.1 (12-18)	1	81 (70%)	60 (20-84)	17.5 (10-48)	116 (100%)	74 (64%)	91 (78%)
El-Shehaby et al. 2021 [13]	273	273 (100%)	Retrospective cohort	74	SS-SRS	12 (9-15)	1	201 (74%)	48 (20-83)	15.5 (10-57)	273 (100%)	87 (32%)	228 (84%)
Goyal-Honavar et al. 2024 [14]	70	70 (100%)	Retrospective cohort	48	SS-SRS	^†^12.5 (±1.2)	1	47 (62%)	^†^46.3 (±11)	^†^12.55 (±5)	26 (81%)	39 (51%)	35 (46%)
Han et al. 2017 (SS) [2]	42	42 (100%)	Retrospective cohort	57.8	SS-SRS	12 (8-14)	1	30 (71%)	66 (28-86)	15.2 (10-48)	NA	14 (33%)	24 (57%)
Han et al. 2017 (HSRS) [2]	28	28 (100%)	Retrospective cohort	50	HSRS	2 FX; 15 (10-16), 3 FX; 18 (15-19), 4 FX; 18 (NA)	2-4	16 (57%)	59 (27-81)	21 (10-54)	NA	8 (28%)	16 (57%)
Haselsberger et al. 2009 [15]	20	20 (100%)	Retrospective cohort	90	Staged-SRS	12 (NA)	2-3	14 (70%)	60.5 (26-73)	33.3 (13-79)	14 (70%)	14 (70%)	14 (70%)
*Hirano et al. 2025 [3]	24	15 (63%)	Retrospective cohort	22	HSRS	28 (21-35)	3/5	12 (50%)	70 (50-88)	14 (2-38)	15 (100%)	13 (54%)	NA
Iwai et al. 2019 [16]	24	24 (100%)	Retrospective cohort	84	Staged-SRS	10 (8-12)	2	15 (63%)	64.5 (20-83)	27.5 (14-49)	24 (100%)	12 (50%)	24 (100%)
*Fatima et al. 2019 [17]	74	59 (80%)	Retrospective cohort	32.8	HSRS/ SS-SRS	24 (14-30)	1-5	39 (53%)	60 (22-92)	16 (10-65)	46 (78%)	33 (44%)	59 (100%)
Oh et al. 2020 [18]	31	31 (100%)	Retrospective cohort	57	HSRS	27.8 (22-27)	3-5	22 (71%)	55 (35-82)	18.9 (11-58)	NA	4 (13%)	31 (100%)
Park et al. 2018 [19]	23	23 (100%)	Retrospective cohort	^†^38	HSRS	18 (15-20)	3-4	17 (74%)	^‡^65 (54-80)	15.1 (10-71)	NA	0 (0%)	23 (100%)
*Pinzi et al. 2023 [20]	166	91 (51%)	Prospective cohort	53	HSRS	25 (NA)	5	121 (73%)	57 (24-87)	14.0 (0.7-126)	166 (100%)	82 (49%)	145 (87%)
Song and Go 2024 [21]	18	18 (100%)	Retrospective cohort	30.5	HSRS	24 (21-30)	3-5	13 (72%)	77.5 (45-90)	14.8 (11-28)	NA	0 (0%)	9 (50%)
Su et al. 2017 [22]	2	2 (100%)	Retrospective cohort	105	Staged-SRS	SI; 18.6 (16-21), SII; 12.4 (11-13)	2	2 (100%)	37.5 (33-42)	45.4 (21-69)	2 (100%)	2 (100%)	2 (100%)
Taori et al. 2025 [23]	112	112 (100%)	Retrospective cohort	106	SS-SRS	12 (10-15)	1	83 (74%)	65 (53-85)	13 (10-24)	112 (100%)	0 (0%)	67 (60%)
*Verma et al. 2023 [24]	70	31 (44%)	Retrospective cohort	36	SS-SRS/ HSRS	14 (8-25)	1-2	28 (40%)	45 (11-75)	25.4 (20-78)	28 (90%)	47 (67%)	NA

Pooled Analysis

**Figure 2:**
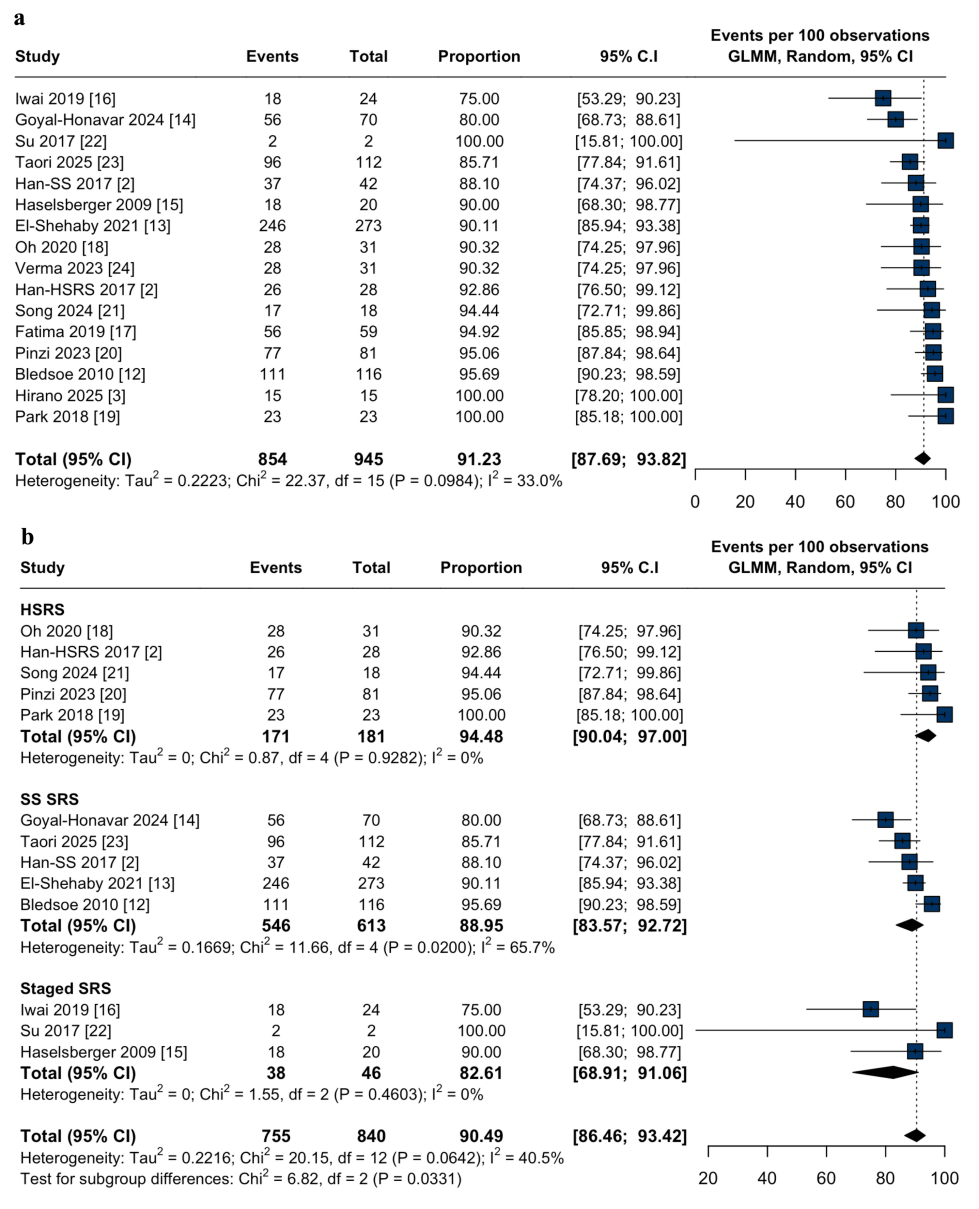
Tumor control after stereotactic radiosurgery for large-volume meningiomas: pooled analysis and subgroup comparison. (a) Forest plot of the pooled proportion of overall tumor control following stereotactic radiosurgery (SRS) for large-volume meningiomas at last follow-up; (b) Forest plot of subgroup analysis comparing hypofractionated (HSRS), single-session (SS-SRS), and staged stereotactic radiosurgery (staged-SRS) for tumor control in large-volume meningiomas. The test for subgroup differences was statistically significant (p = 0.03). CI: confidence interval, GLMM: generalized linear mixed model.

**Figure 3:**
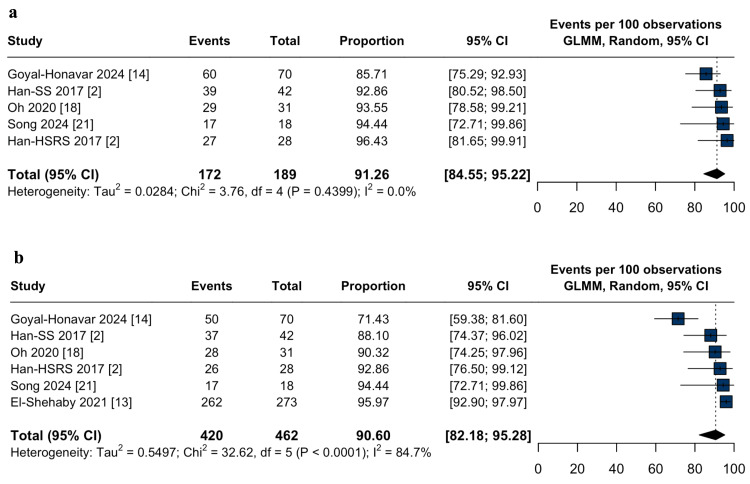
Progression-free survival after stereotactic radiosurgery for large-volume meningiomas: pooled three-year and five-year estimates. (a) Forest plot of the pooled three-year progression-free survival (PFS) following stereotactic radiosurgery (SRS) for large-volume meningiomas; (b) Forest plot of the pooled five-year progression-free survival (PFS) following stereotactic radiosurgery (SRS) for large-volume meningiomas. CI: confidence interval, GLMM: generalized linear mixed model.

**Figure 4:**
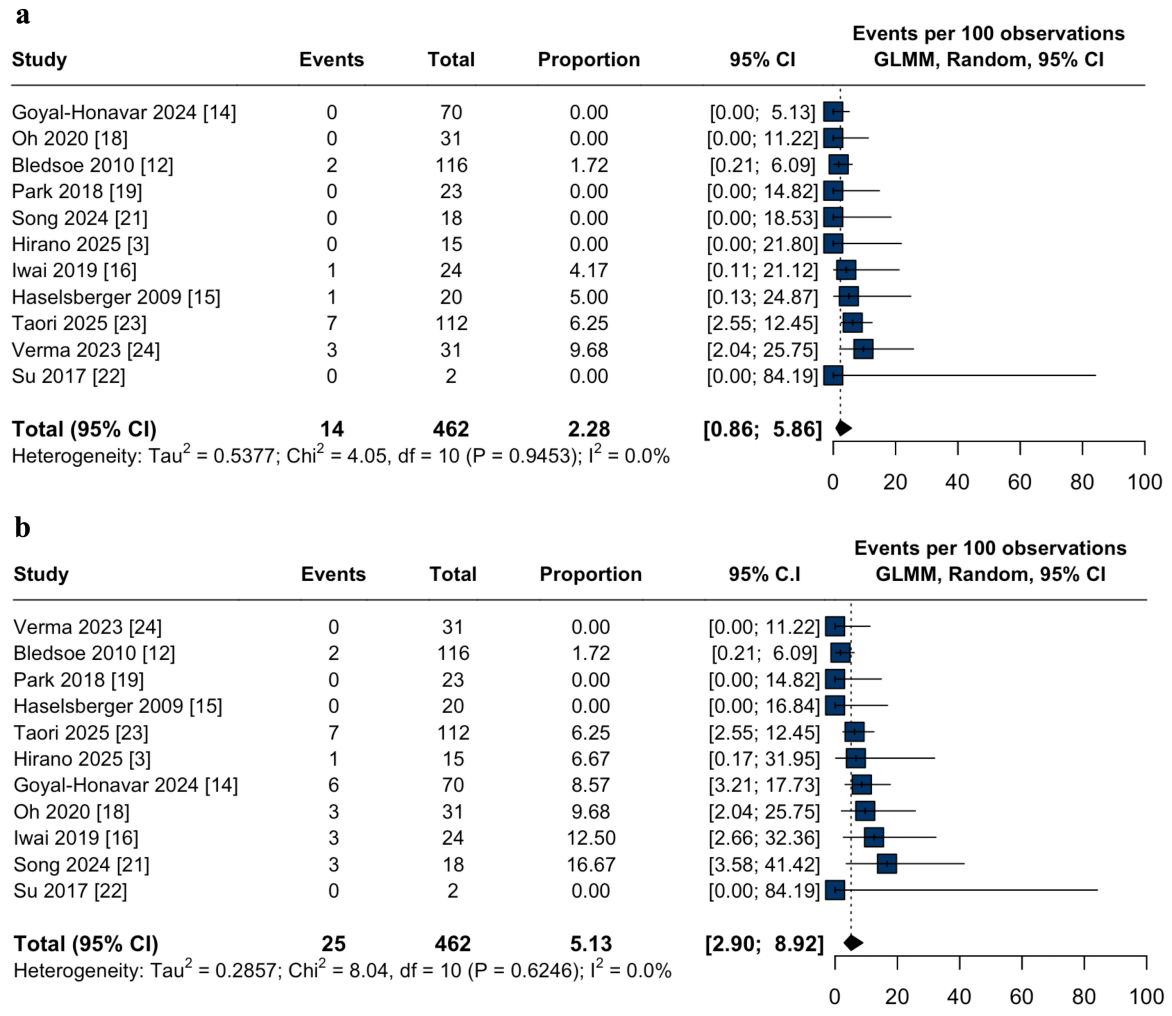
Repeat radiation therapy and salvage surgery after stereotactic radiosurgery for large-volume meningiomas: pooled estimates. (a) Forest plot of the pooled proportion of patients requiring repeat radiation therapy following stereotactic radiosurgery (SRS) for large-volume meningiomas; (b) Forest plot of the pooled proportion of salvage craniotomy with tumor resection following stereotactic radiosurgery (SRS) for large-volume meningiomas. CI: confidence interval, GLMM: generalized linear mixed model.

Efficacy outcomes: The overall tumor control rate was 91.2% (95% CI: 87.6-93.8%; I² = 33%; Figure 2a). Subgroup analyses demonstrated higher tumor control with HSRS (94.4%; 95% CI: 90.0-97.0%; I² = 0.0%; p = 0.03; Figure 2b) compared to single-session (88.9%; 95% CI: 83.5-92.7%; I² = 65.7%; p = 0.03; Figure 2b) and staged-SRS (82.6%; 95% CI: 68.9-91.0%; I² = 0.0%; p = 0.03; Figure 2b). Progression-free survival at three and five years was 91.2% (95% CI: 84.5-95.2%; I² = 0.0%; Figure 3a) and 90.6% (95% CI: 82.1-95.2%; I² = 84.7%; Figure 3b), respectively. Repeat radiation therapy was required in 2.2% (95% CI: 0.8-5.8%; I² = 0.0%; Figure 4a), and salvage craniotomy with tumor resection following SRS was performed in 5.1% (95% CI: 2.9-8.9%; I² = 0.0%; Figure 4b). 

**Figure 5:**
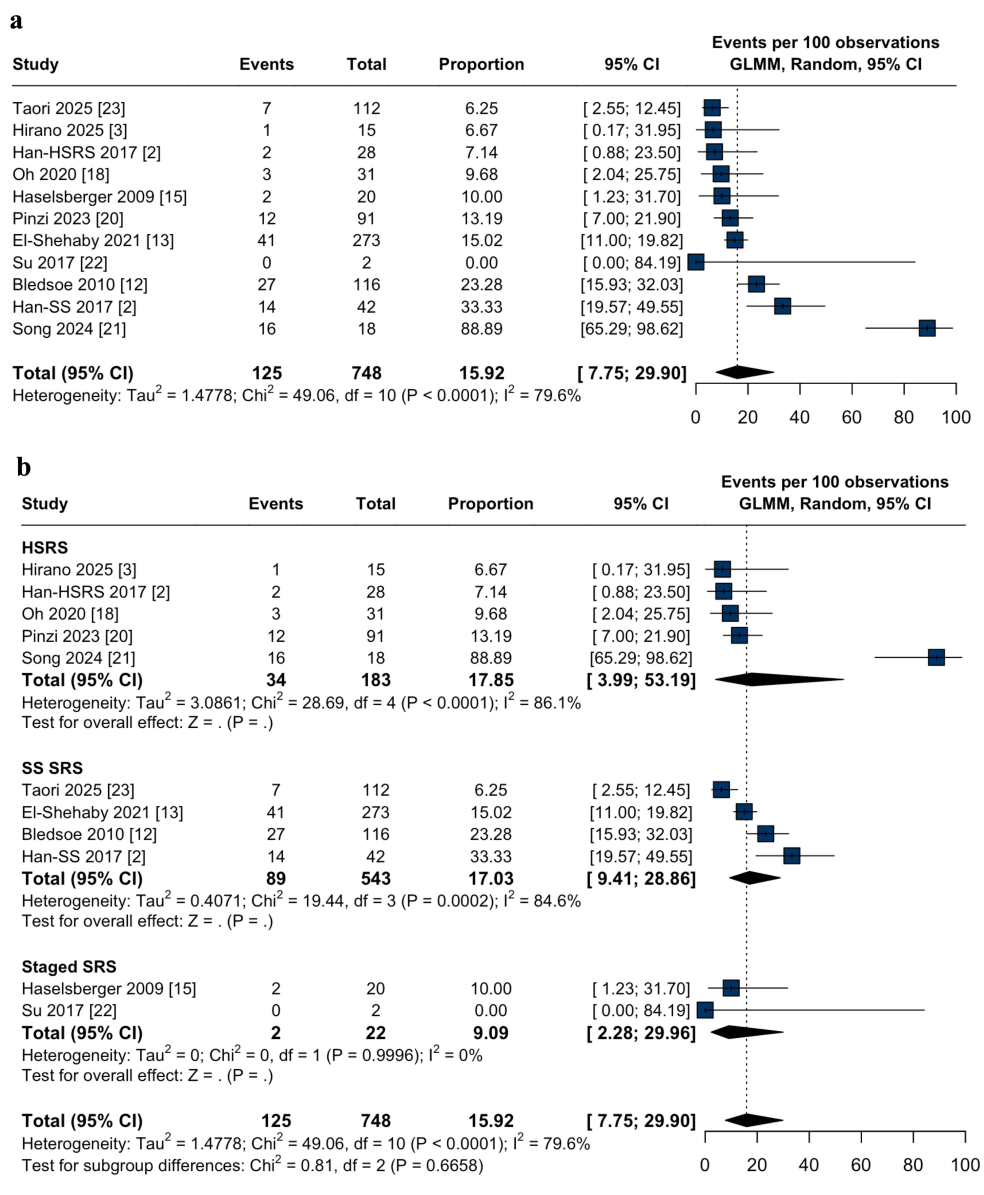
Adverse radiation effects after stereotactic radiosurgery for large-volume meningiomas: pooled analysis and subgroup comparison. (a) Forest plot of the pooled proportion of adverse radiation effects (AREs) following stereotactic radiosurgery (SRS) for large-volume meningiomas; (b) Forest plot of subgroup analysis comparing hypofractionated (HSRS), single-session (SS-SRS), and staged stereotactic radiosurgery (staged-SRS) for adverse radiation effects in large-volume meningiomas. The test for subgroup differences was not statistically significant (p = 0.67). CI: confidence interval, GLMM: generalized linear mixed model.

Safety outcomes: Adverse radiation effects (AREs) were reported in 15.9% of cases (95% CI: 7.7-29.9%; I² = 79.6%; Figure 5a). Subgroup analysis revealed comparable pooled rates of AREs among patients treated with HSRS (17.8%; 95% CI: 3.9-53.1%; I² = 86.1%; Figure 5b), single-session (17.0%; 95% CI: 9.4-28.8%; I² = 84.6%; Figure 5b) and staged-SRS (9.0%, 95% CI: 2.2-29.9%; I² = 79.6%; Figure 5b), with no statistically significant difference between groups (p = 0.67). The HSRS subgroup exhibited substantial heterogeneity, largely driven by an outlier event rate reported in one study. Sensitivity analysis excluding Song et al. [21] resulted in a reduced pooled incidence of 10.9% (95% CI: 6.9-16.6%; I² = 0.0%; Appendix A) and eliminated heterogeneity. 

**Figure 6:**
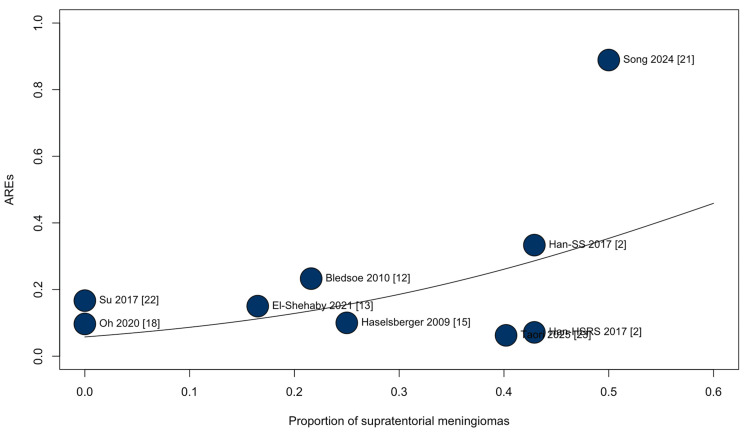
Adverse radiation effects after stereotactic radiosurgery for large-volume meningiomas: meta-regression by tumor location. Mixed-effects meta-regression of adverse radiation effects (AREs) according to the cohort-level proportion of supratentorial meningiomas. Cohorts with a greater proportion of supratentorial meningiomas demonstrated increased rates of adverse radiation effects (β = 4.38; 95% CI: 1.97–6.79; p = 0.0004).

To explore whether anatomical distribution contributed to variability in toxicity, an exploratory mixed-effects meta-regression including nine studies assessed the relationship between the study-level proportion of supratentorial meningiomas and the logit-transformed rate of AREs. Cohorts with a higher proportion of supratentorial tumors demonstrated significantly higher ARE rates (β = 4.38; 95% CI 1.97-6.79; p = 0.0004; Figure 6), although residual heterogeneity remained substantial (τ² = 1.37; I² = 90.2%). This suggests that tumor location may partially account for between-study differences in reported toxicity, but the finding should be interpreted cautiously given the study-level nature of the analysis.

**Figure 7:**
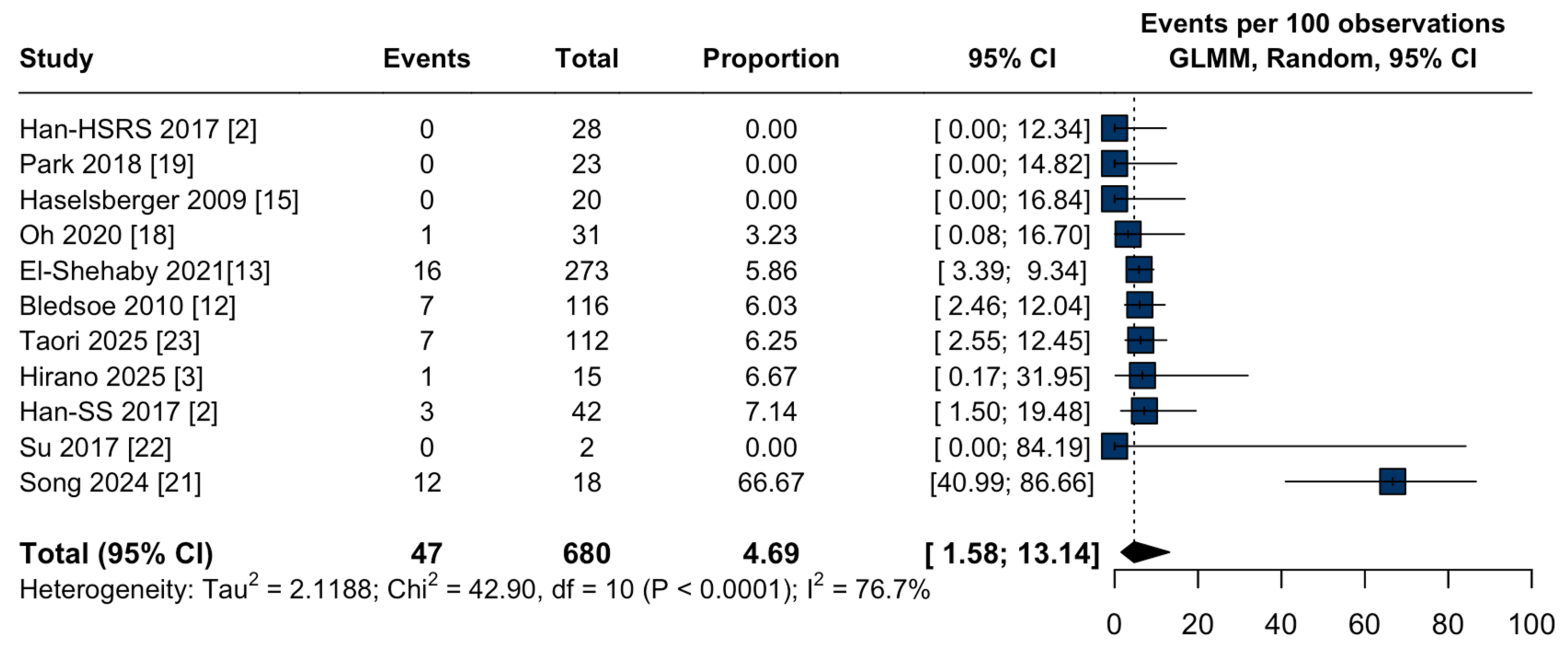
Symptomatic peritumoral edema after stereotactic radiosurgery for large-volume meningiomas. Forest plot of the pooled proportion of symptomatic peritumoral edema following stereotactic radiosurgery (SRS) for large-volume meningiomas. CI: confidence interval, GLMM: generalized linear mixed model.

**Figure 8:**
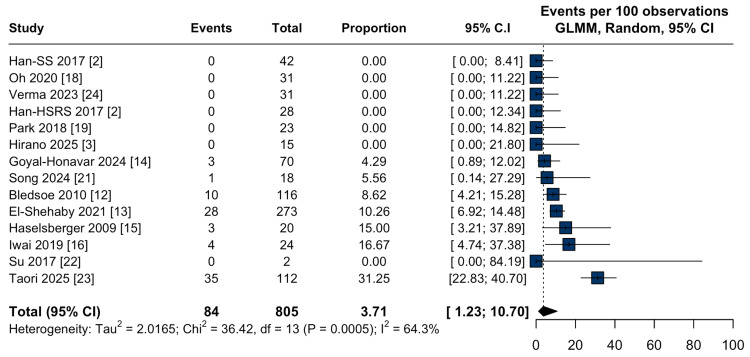
All-cause mortality after stereotactic radiosurgery for large-volume meningiomas. Forest plot of the pooled proportion of all-cause mortality following stereotactic radiosurgery (SRS) for large-volume meningiomas. CI: confidence interval, GLMM: generalized linear mixed model.

**Figure 9:**
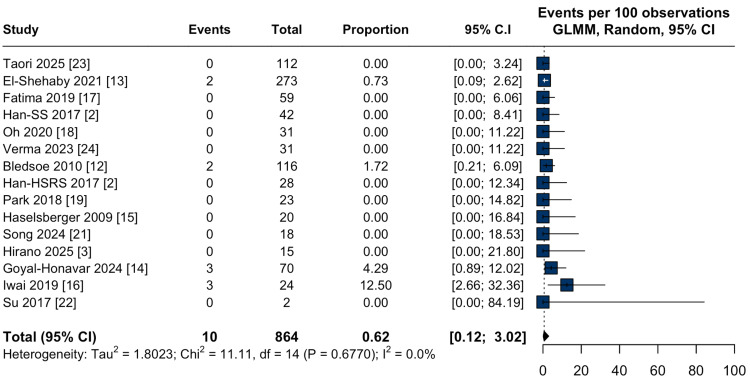
Tumor-related mortality after stereotactic radiosurgery for large-volume meningiomas. Forest plot of the pooled proportion of tumor-related mortality following stereotactic radiosurgery (SRS) for large-volume meningiomas. CI: confidence interval, GLMM: generalized linear mixed model.

Symptomatic peritumoral edema occurred in 4.6% (95% CI: 1.5-13.1%; I² = 76.7%; Figure 7). All-cause mortality was 3.7% (95% CI: 1.2-10.7%; I² = 64.3%; Figure 8), and tumor-related mortality was rare, with a pooled incidence <1% (95% CI: 0.1-3.0%; I² = 0.0%; Figure 9). 

Sensitivity Analysis

Leave-one-out sensitivity analyses were conducted for primary outcomes and those exhibiting high heterogeneity to assess the influence of individual studies on pooled estimates. For overall tumor control, omission of Goyal-Honavar et al. [14] reduced heterogeneity to 13.1% (Appendix B). For progression-free survival at five years, omission of the same study reduced heterogeneity to 21.8% (Appendix C). For adverse radiation effects, no single study meaningfully altered heterogeneity in the overall cohort (Appendix D); however, within the HSRS subgroup, omission of Song et al. [21] reduced heterogeneity to 0.0% (Appendix A). Omission of Song et al. [21] also reduced heterogeneity to 0.0% for symptomatic peritumoral edema (Appendix E). In the analysis of all-cause mortality, omission of Taori et al. [23] similarly reduced heterogeneity to 0.0% (Appendix F). 

Quality Assessment and Risk of Publication Bias Assessment

Based on the ROBINS-I tool, our analysis identified a serious risk of bias in 14 studies, mostly due to confounding. A moderate risk of bias was identified in 1 study. (Appendix G). Funnel plot analyses for efficacy and safety outcomes revealed no indication of publication bias (Appendix H and Appendix I). These findings were corroborated by Egger's regression test, which showed no significant evidence of bias for overall tumor control (intercept = 0.93; 95% CI: −0.26 to 2.12; p = 0.15) or adverse radiation effects (intercept = −0.02; 95% CI: −2.55 to 2.52; t = −0.01; p = 0.99).

Discussion

We conducted a systematic review and meta-analysis to evaluate the efficacy and safety of stereotactic radiosurgery (SRS) for large-volume meningiomas (LVMs), defined as tumors measuring ≥10 cm³. Fifteen studies comprising a total of 955 patients were included in the quantitative synthesis. The principal findings of this analysis are as follows: (1) SRS is associated with high overall tumor control, in both upfront and adjuvant settings; (2) the incidence of adverse radiation effects (AREs) is moderate; (3) serious complications such as symptomatic peritumoral edema and treatment-related mortality are infrequent; and (4) hypofractionated SRS (HSRS) appears to provide superior tumor control compared to single-session and staged SRS, while maintaining a comparable safety profile.

Surgical resection remains the primary treatment for most intracranial meningiomas, particularly when gross total resection (GTR) is feasible and morbidity is acceptable. However, resection of large tumors, especially those in skull base or eloquent locations, is often limited by the risk of cranial neuropathies, cerebrospinal fluid leaks, and incomplete resection due to neurovascular encasement [25-27]. These challenges are further compounded in recurrent cases, where reoperation is associated with lower rates of GTR and higher morbidity, as demonstrated by Magill et al. [28]. In such cases, SRS offers a minimally invasive alternative or adjunct that can achieve durable tumor control while minimizing treatment-related morbidity. Its efficacy in small- to medium-sized lesions is well established [29-31], but emerging data now support its use in larger tumors, particularly when hypofractionated or staged delivery is employed [5,25,32]. Notably, advances in imaging, dose planning, and patient immobilization have markedly improved the precision and safety of SRS in anatomically complex locations [1].

A recent meta-analysis by Hajikarimloo et al. [5] examined SRS in the context of large intracranial meningiomas using broader volumetric and dimensional inclusion criteria (tumors >8 cm³ or >2.5 cm in diameter), reporting pooled tumor control and ARE rates of 92% and 16%, respectively. While these findings support the general feasibility of SRS in larger tumors, the analysis synthesized data from a clinically heterogeneous population and did not stratify outcomes by tumor volume. As such, its applicability to the highest-risk surgical candidates may be limited. In contrast, our meta-analysis applies a strict volumetric threshold of ≥10 cm³, providing outcome estimates that are more representative of anatomically complex or surgically inaccessible lesions. Pooled tumor control (91.2%) and ARE (15.9%) suggest potential disease control with measurable toxicity; however, given serious ROBINS-I concerns in most studies and between-study heterogeneity, these figures are contextual and should be interpreted with caution.

Subgroup analysis further demonstrated that HSRS was associated with the highest tumor control rate (94.4%), outperforming single-session (88.9%) and staged-SRS (82.6%), with statistically significant differences and no interstudy heterogeneity (p = 0.03; I² = 0.0%). In contrast, the analogous subgroup analysis in the aforementioned study did not achieve statistical significance and was accompanied by higher heterogeneity (p = 0.21; I² = 34.3%). Rather than duplicating prior findings, our results extend the existing literature by delineating modality-specific outcomes within a narrowly defined, high-risk population. Notably, this meta-analysis incorporates the largest pooled cohort of patients with tumors ≥10 cm³ to date, thereby enhancing statistical power and improving the precision and generalizability of subgroup comparisons.

Given the established association between tumor volume and radiosurgical toxicity [12,32], we also evaluated AREs across SRS modalities. The overall pooled incidence was 15.9%, with no statistically significant differences among HSRS (17.8%), single-session (17.0%), and staged-SRS (9.0%) (p = 0.66). Radionecrosis was not consistently reported as a distinct endpoint across the included studies; therefore, its apparent absence in pooled analyses should be interpreted cautiously, as underreporting and variable toxicity definitions may obscure both local and distant radiation-induced effects, underscoring the need for prospective studies with standardized toxicity assessment. Substantial heterogeneity was observed within the HSRS subgroup (I² = 86.1%), largely attributable to a single outlier study with an elevated event rate [21]. Leave-one-out sensitivity analysis excluding this study reduced the pooled estimate to 10.9% and resolved heterogeneity (I² = 0.0%), suggesting that the safety profile of HSRS may be more favorable and consistent than initially perceived. In comparison, the prior meta-analysis [9] reported an AREs rate of 37% for HSRS, though statistical significance was achieved only under fixed-effects modeling. These findings underscore the importance of prospective, standardized studies to clarify modality-specific toxicity risks and inform patient-centered treatment planning.

Exploratory meta-regression showed that cohorts with a greater proportion of supratentorial meningiomas reported higher rates of adverse radiation effects (β = 4.38; p = 0.0004). This relationship should be interpreted as hypothesis-generating, as it reflects study-level data and cannot account for dose selection, margin definition, edema prophylaxis, or follow-up duration, and substantial residual heterogeneity persisted. Even with these limitations, the finding suggests that anatomical compartments may affect radiosurgical tolerance in large-volume meningiomas and should be considered when estimating toxicity risk and planning treatment.

Our findings are further supported by the most recent consensus guidelines from the International Stereotactic Radiosurgery Society (ISRS) [25], which endorse the use of SRS for WHO Grade I meningiomas in patients who are suboptimal surgical candidates, particularly when tumors are residual, recurrent, or located near eloquent structures. For larger lesions, the ISRS recommends hypofractionated regimens (25 Gy in 5 fractions) to optimize tumor control while minimizing treatment-related morbidity. The superior local control observed with HSRS in our volume-stratified cohort aligns with these recommendations and contributes additional granularity to inform clinical decision-making in complex cases.

Limitations

Our study has several limitations. First, the lack of randomized controlled trials among the included studies restricts causal inference, as all data were derived from observational designs. Second, the single-arm nature of this meta-analysis precludes direct comparison with other treatment modalities. Nonetheless, subgroup analyses across different radiosurgical strategies were conducted to explore potential outcome differences. Additionally, although several included studies reported extended follow-up, long-term outcomes remain inconsistently reported, limiting the ability to fully assess the durability of tumor control and the incidence of late radiation-related toxicity. Finally, substantial heterogeneity was present in select outcomes, which we addressed through leave-one-out sensitivity analyses to evaluate result robustness.

## Conclusions

For meningiomas ≥10 cm³, stereotactic radiosurgery may be associated with high tumor control and an acceptable toxicity profile. Hypofractionated stereotactic radiosurgery (HSRS) appears to offer higher tumor control while maintaining comparable toxicity. Exploratory analyses suggest that supratentorial tumor location may be associated with a higher risk of adverse radiation effects. These findings should be considered hypothesis-generating; prospective studies with standardized endpoints and longer follow-up are needed to establish the durability of tumor control and further refine patient selection and dose-fractionation protocols.

## Figures and Tables

**Figure 10:**
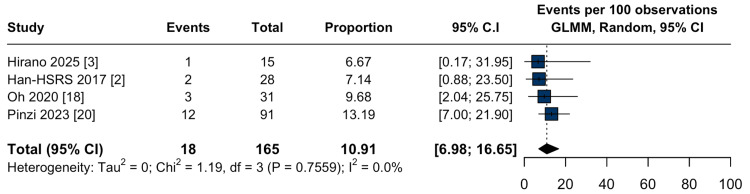
Forest plot for leave-one-out sensitivity analysis of adverse radiation effects (AREs) in the hypofractionated stereotactic radiosurgery (HSRS) subgroup, excluding Song et al. CI: confidence interval, GLMM: generalized linear mixed model.

**Figure 11:**
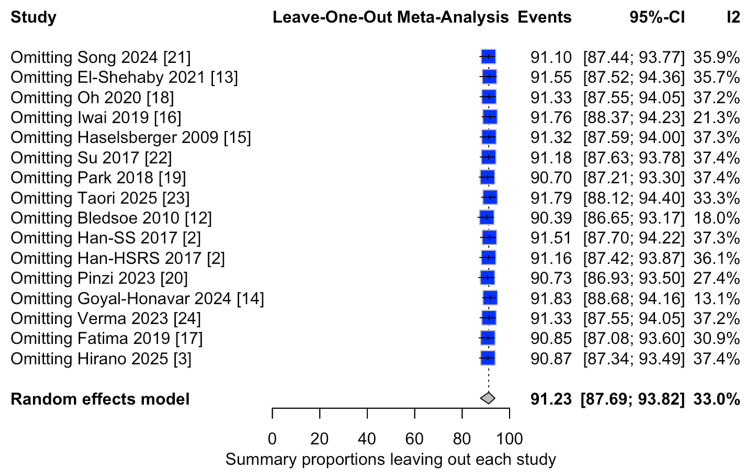
Leave-one-out analysis for overall tumor control. CI: confidence interval.

**Figure 12:**
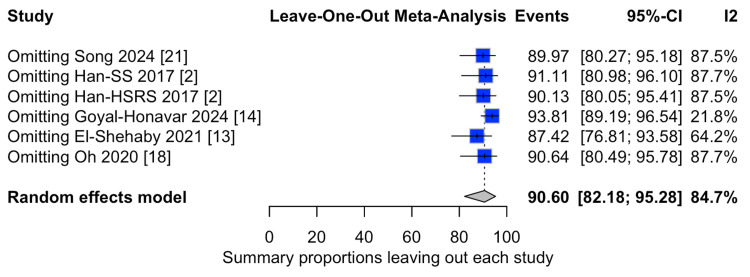
Leave-one-out analysis for progression-free survival at five years. CI: confidence interval.

**Figure 13:**
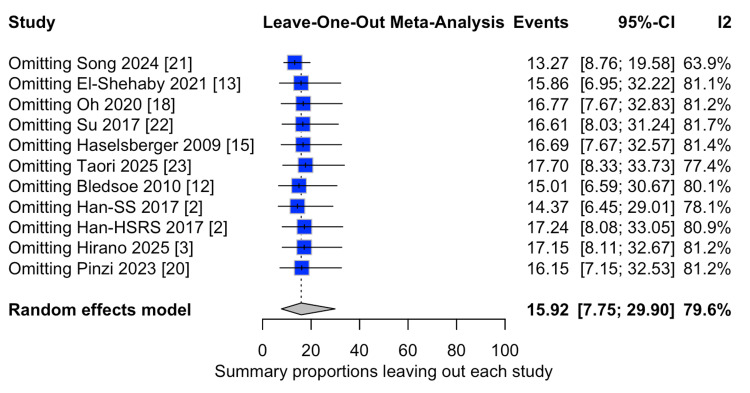
Leave-one-out analysis for adverse radiation effects. CI: confidence interval.

**Figure 14:**
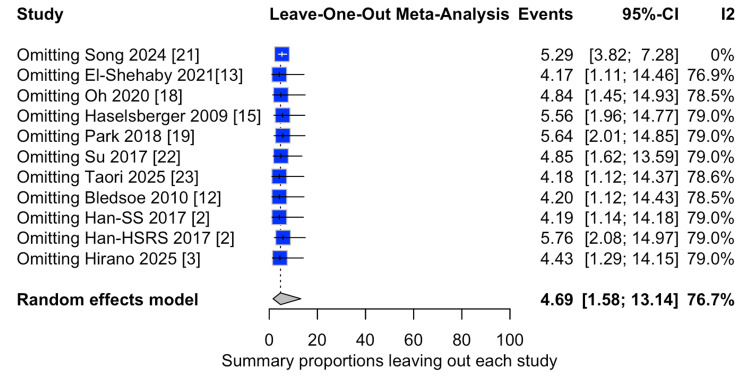
Leave-one-out analysis for symptomatic peritumoral edema. CI: confidence interval.

**Figure 15:**
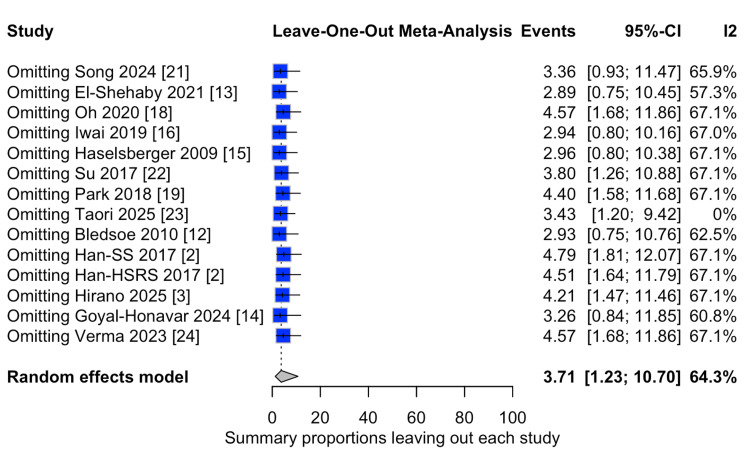
Leave-one-out analysis for all-cause mortality. CI: confidence interval.

**Figure 16:**
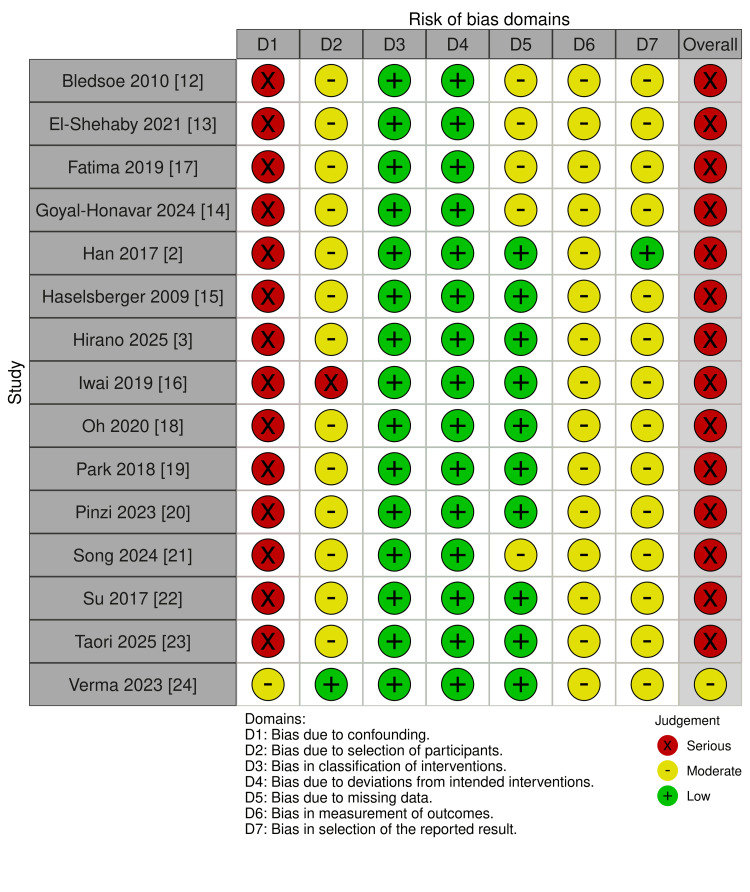
Risk of bias assessment using the ROBINS-I tool.

**Figure 17:**
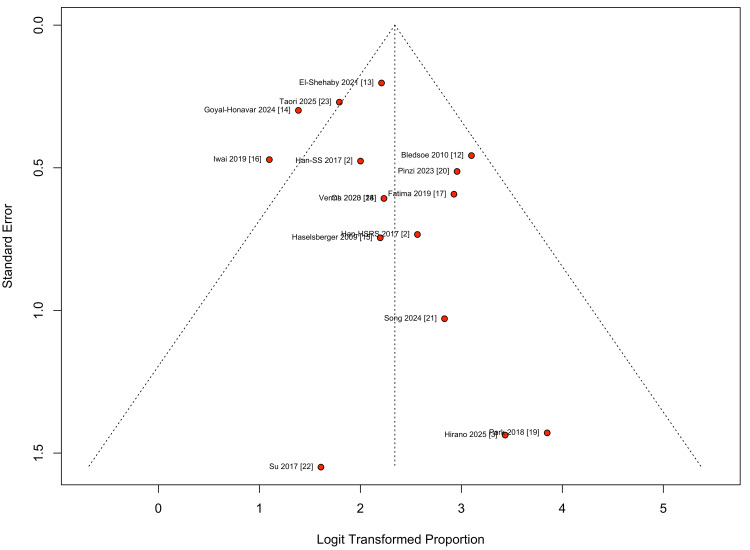
Funnel plot assessing publication bias for overall tumor control.

**Figure 18:**
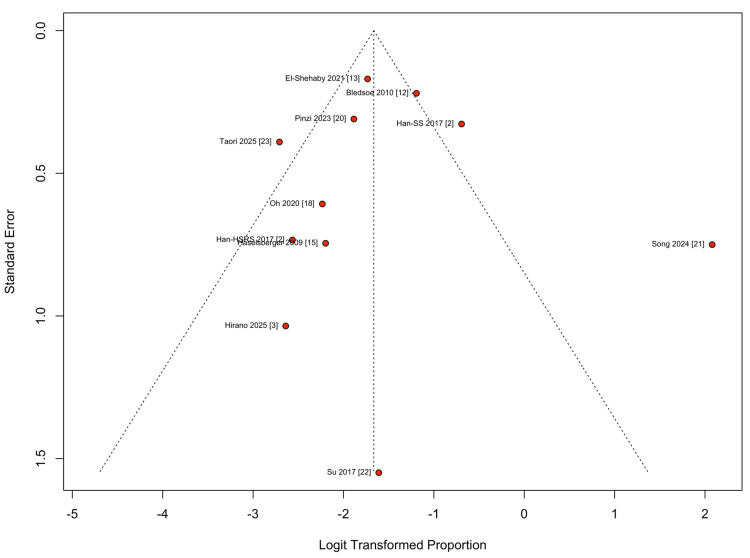
Funnel plot assessing publication bias for adverse radiation effects.

## Appendices

Appendix A

Appendix B 

Appendix C 

Appendix D 

Appendix E 

Appendix F 

Appendix G 

Appendix H 

Appendix I 
